# A proximity-based graph clustering method for the identification and application of transcription factor clusters

**DOI:** 10.1186/s12859-017-1935-y

**Published:** 2017-11-29

**Authors:** Maxwell Spadafore, Kayvan Najarian, Alan P. Boyle

**Affiliations:** 1 University of Michigan Medical School, 1301 Catherine, Ann Arbor, 48109-5624 USA; 2 University of Michigan Department of Computational Medicine and Bioinformatics, 100 Washtenaw Avenue, Ann Arbor, 48109 USA; 3 University of Michigan Medical School Department of Emergency Medicine, 1500 E Medical Center Drive, Ann Arbor, 48109 USA; 4 University of Michigan Department of Genetics, 1241 E Catherine, Ann Arbor, 48109 USA

**Keywords:** Transcription factors, Graph theory, Graph clustering, Network analysis, TF clusters, Genome regulation

## Abstract

**Background:**

Transcription factors (TFs) form a complex regulatory network within the cell that is crucial to cell functioning and human health. While methods to establish where a TF binds to DNA are well established, these methods provide no information describing how TFs interact with one another when they do bind. TFs tend to bind the genome in clusters, and current methods to identify these clusters are either limited in scope, unable to detect relationships beyond motif similarity, or not applied to TF-TF interactions.

**Methods:**

Here, we present a proximity-based graph clustering approach to identify TF clusters using either ChIP-seq or motif search data. We use TF co-occurrence to construct a filtered, normalized adjacency matrix and use the Markov Clustering Algorithm to partition the graph while maintaining TF-cluster and cluster-cluster interactions. We then apply our graph structure beyond clustering, using it to increase the accuracy of motif-based TFBS searching for an example TF.

**Results:**

We show that our method produces small, manageable clusters that encapsulate many known, experimentally validated transcription factor interactions and that our method is capable of capturing interactions that motif similarity methods might miss. Our graph structure is able to significantly increase the accuracy of motif TFBS searching, demonstrating that the TF-TF connections within the graph correlate with biological TF-TF interactions.

**Conclusion:**

The interactions identified by our method correspond to biological reality and allow for fast exploration of TF clustering and regulatory dynamics.

**Electronic supplementary material:**

The online version of this article (doi:10.1186/s12859-017-1935-y) contains supplementary material, which is available to authorized users.

## Background

Transcription factors (TFs) are proteins that specifically regulate the transcription of DNA to RNA within the cell. There are an estimated 1300 human TFs, and they can act as suppressors or enhancers of transcription in a variety of ways, either directly, by binding and remodeling the structure of DNA itself, or indirectly, by binding to and influencing other TFs [1]. The transcriptional regulation brought about by TFs is crucial to the health of the cell and of the organism, with transcriptional regulation central to cell cycle control [2], cell homeostasis [3], and cell differentiation [4]. The consequences of TF failure can be severe, with one-third of human developmental disorders attributed to TF errors [5]. As such, it is critical to understand the complex regulatory network that TFs create.

While chromatin immunoprecipitation and sequencing (ChIP-seq) assays [6, 7] and motif analysis [8, 9] can be used to determine *where* TFs bind DNA, neither provides information on *how* the TFs bind. TFs tend to cooperatively bind the genome as large complexes, or clusters, binding to the DNA, one another, or both [10, 11]. In these situations, one or more “anchor” TFs bind the DNA directly, and then other TFs bind the anchors rather than the DNA. This creates a combinatorial problem, wherein a given anchor TF may be bound by several different other TFs depending on time, cellular conditions, etc., and a given association (non-anchor) TF may bind several different anchor TFs. This “second dimension” of TF binding is largely unexplored, and it may even explain part of the discrepancy between motif sequence quality among TFs. Given that anchor TFs bind the DNA directly, they are expected to have high-quality motif sequences. The associating TFs, however, would be expected to have poorer, degenerate motif sequences due to the fact that they may not directly bind the DNA and may be associated with different anchor TFs under different conditions.

Understanding the makeup of TF complexes, then, would allow for better utilization of motif sequences in TFBS prediction as well as promote further understanding of the TF regulatory framework of the genome in general. Neither ChIP-seq nor motif sequences provide TF complex information on their own, however, so various algorithmic and data integration approaches have been taken to discover TF clusters. These methods can each be roughly assigned to one of three categories: experimental, similarity, and proximity.

Experimental TF complex investigations focus on discovering and characterizing one complex at a time (see [12–14] as representative examples). While these methods use accurate in vitro or in vivo assays, they are low-throughput and narrow, unable to identify interactions beyond those their assays search for.

Similarity-based methods, such as those in [15] and [16], exploit the inherent basis of PWMs as simple matrices. They assume that TFs which bind similar sequences are likely to bind at the same locations and interact with one another, and they calculate similarity scores between individual TFs’ PWMs and cluster based on these scores. These methods have the advantage of not needing PWMs to be aligned to the genome first, but they inherently miss TF-TF interactions *not* based on affinity for the same sequence, such as the anchor-association paradigm described above.

Finally, proximity-based methods, including [17, 18], and [11], use TFBS data (either putative, from motifs, or experimental, from ChIP-seq) to cluster TFs based on their co-occurrence in close proximity. They make the assumption that TFs which interact will inherently appear with one another more often than the genomic background. Because they use proximity data rather than PWMs, they are able to cluster TFs which possibly interact but have differing PWMs. However, the methods in [17] and [18] are not applied directly to cluster exploration, instead focusing on TFBS density and association with other regulatory elements, respectively. Additionally, while the method in [11] does focus directly on TF clustering, it requires supplementary input from a mass-spectroscopy dataset.

From the above, we can see that the TF regulatory framework is highly complex, including not only a large number of TFs but a myriad of interactions between them. Neither ChIP-seq nor motif searching can identify TF interactions on their own, and existing cluster-finding methods are either limited in scope, unable to detect non-similarity relationships, or not applied to TF-TF interactions. As a result, there is a need for a proximity-based clustering method which focuses on discerning and exploring TF-TF clusters and interactions.

Here, we demonstrate the usefulness of such a proximity-based graph clustering method for the identification, exploration, and application of TF-TF clusters. By transforming TF co-occurrence data into a graph which is then clustered using the Markov Clustering Algorithm, our method putatively identifies all of the TF clusters within a given cell type in one pass and requires only two parameters to function. Clusters can be produced using either ChIP-seq or motif TFBS data as inputs, and we test our method using 111 ChIP-seq experiments and 585 TF PWMs. We show that the returned clusters agree with known, experimentally confirmed TF-TF interactions. We use an empirical method to set the false positive rate (FPR) and show that clustering performance remains stable even at very low FPRs. We also show that our method’s clusters incorporate more information than similarity alone, demonstrating that connection in our method’s graph is not highly correlated with PWM similarity. Finally, we provide an example of utilizing the graph information to significantly improve the accuracy of TFBS searching using motif sequences.

## Methods

### Method overview

Our method exploited the simple fact that TFs which often interact must have binding sites, as labeled by ChIP-seq or detected by motif searching, near one another, developing a graph with edges weighted by a normalized TF-TF co-occurrence score. To calculate this score for each TF-TF pair, we first created *co-occurrence matrices*, one for each TF, that contained the neighboring TFs at each TFBS of the given transcription factor. These co-occurrence matrices were then transformed to create a series of normalized *co-occurrence vectors* which contained the co-occurrence frequencies for a set of potential TF-TF interactions. These vectors were assembled into the adjacency matrix.

If the adjacency matrix was used for clustering, its edges were first filtered by selecting an FPR and removing edges with weight lower than a threshold empirically determined to uphold the selected FPR. Markov clustering was then performed on the filtered matrix. It is important to note that the resulting clusters partitioned TFs, rather than individual TFBSs, producing results similar to that of a protein-protein interaction database, except specific to a given cell type and based on genomic regulation rather than general protein interaction.

**Fig. 1:**
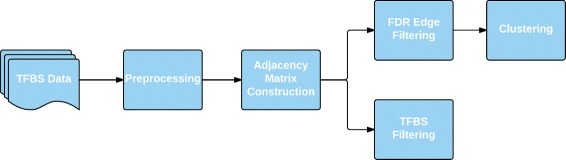
Overview of the method

The graph was also used to filter putative TFBSs in order to increase the precision and recall of motif searching, exploiting the fact that motif matches are less likely to be false positives if they fall near the motif matches of their highly co-occurring counterparts. In this case, no FDR filtering was done. Instead, summed edge weights were used to threshold and remove putative TFBSs which do not fit the co-occurrence profile in the graph. Figure 1 provides a flowchart overview of our method.

### Data sources and preprocessing

We used two datasets in our analysis. The first, the *ChIP-seq dataset*, used ChIP-seq data for 111 TFs in the cell type K562 from the Encyclopedia of DNA Elements (ENCODE) Project [19]. Each ChIP-seq experiment’s data was uniformly processed by ENCODE to identify the location of ChIP-seq “peaks”, or ChIP-seq identified TFBSs, within the genome. The TFBSs from the separate experiments were assembled and sorted into one large dataset containing over 1.4 million TFBSs. We chose the K562 cell type because it contains the most ChIP-seq data of all cell types within ENCODE. Because TF-TF interactions change between cell types (and in many ways define their different behaviors), the clusters produced by our method were therefore specific to K562.

The second dataset was the *ENCODE-motif dataset*. To develop ENCODE-motif, Kheradpour and Kellis characterized, categorized, and discovered motifs using the ENCODE ChIP-seq experiments, and they provide a collection of genomic motif match locations (putative TFBSs) for every motif used in their analysis [20]. This collection contains over 144 million putative TFBSs across 585 transcription factors, and was used in our analysis for clustering of motif-based TFBSs as well as to demonstrate putative TFBS filtering. Kheradpour and Kellis discovered motifs as well as characterized known motifs for their analysis; the former were excluded to focus only on pre-established motifs as well as to reduce the size of the dataset somewhat to 124 million putative TFBSs. To reduce its memory requirements, the dataset was divided into 100 segments and one of every four segments was selected, leaving a final total of 31.4 million putative TFBSs analyzed. If any transcription factor within the ENCODE-motif dataset was represented by multiple motif PWMs, we considered each PWM equivalent, performing our clustering and analysis at the TF level rather than the PWM level.

### Construction of the adjacency matrix

To construct the adjacency matrix, we first constructed a co-occurrence matrix for each TF in the dataset. To do so, we:

Let *T* be the set of all TFs.

Let *B* be the set of all TFBSs.

Let *B*_*ti*_ be TFBS *i* in the subset of *B* encompassing only the TFBSs of TF *t*.

Let *f*(*b*∈*B*,*t*∈*T*)=0 if TF *t* has no binding sites within 1000 bp of TFBS *b*, and 1 otherwise.

Then the co-occurrence matrix was an *n*×*m* binary matrix such that

$$\mathbf{M}_{t \in T} = f(B_{t},T) = \left(\begin{array}{lll} f(B_{t1},T_{1}) & \hdots & f(B_{tm},T_{1}) \\ \vdots & \ddots & \vdots \\ f(B_{t1},T_{n}) & \hdots & f(B_{tm},T_{n}) \end{array}\right) $$

where *n* is the length of *T* and *m* is the length of *B*_*t*_.

**Fig. 2:**
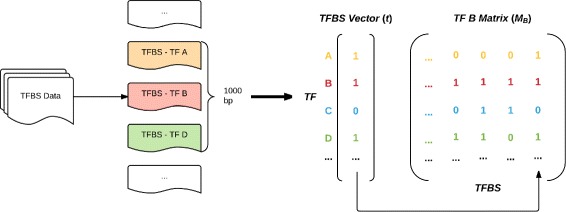
The construction of co-occurrence matrices. For each TFBS of a given TF, the other TFs within a 1000 base-pair window are recorded in that TF’s co-occurrence matrix. The amount of co-occurrence is inversely proportional to the sparsity of the rows; in the above example, TF B is most highly associated with TF D and least associated with TF A

These matrices represent the raw co-occurrence of TFs with one another along the genome; each entry states whether a given TF was found to co-occur (appear within 1000 bp) with another at a particular TFBS. Figure 2 illustrates the construction of these matrices.

Next, a vector **f**_*t*_ was produced for each TF *t*∈*T* such that

$$\mathbf{f}_{t} = \left(\begin{array}{c} \sum_{j=1}^{m} \mathbf{M}_{t1j} \\ \vdots \\ \sum_{j=1}^{m} \mathbf{M}_{tnj} \end{array}\right)m^{-1}$$

**f**_**t**_ was therefore the row means of *M*_*t*_ for each TF *t*. Because *M*_*t*_ is a binary matrix, this produced a vector of co-occurrence frequencies, where each element represented the fraction of TF *t* TFBSs where a given TF was found in close proximity. These frequencies, however, were subject to skew due to the *overall* genomic binding frequencies of their respective TFs. The TF CTCF, for example, binds the genome very frequently, with entire databases devoted to its binding sites, while others, such as GTF2B, bind more rarely [21]. Thus, it is relatively more “important” if GTF2B binds in close proximity to a given TF than CTCF due to CTCF being more prevalent in the background. To account for this, each vector **f**_*t*_ was normalized such that

$${\mathbf{f}^\prime}_{t} = \mathbf{f}_{t} - \mathbf{f}_{\mathbf{all}} $$

with

$$\mathbf{f}_{\mathbf{all}} = \left(\begin{array}{c} \sum_{t \in T} \sum\nolimits_{j} \mathbf{M}_{t1j} \\ \vdots \\ \sum_{t \in T} \sum_{j} \mathbf{M}_{{tnj}} \end{array}\right)w^{-1} $$

where **f** ^′^_*t*_ is the normalized co-occurrence frequency vector, *w* is the length of *B*, and **f**_**all**_ is the *overall* frequency vector - the row mean of all of the *M*_*t*_ matrices concatenated on the horizontal axes. **f**_**all**_ was similar to each **f**_**t**_ matrix, except while each element in **f**_**t**_ represented the co-binding frequencies of a particular TF with TF *t*, each element in **f**_**all**_ represented the binding frequency of a particular TF to the genome overall. Using the example above, we would expect GTF2B to have a lower entry in **f**_**all**_ than CTCF.

**Fig. 3:**
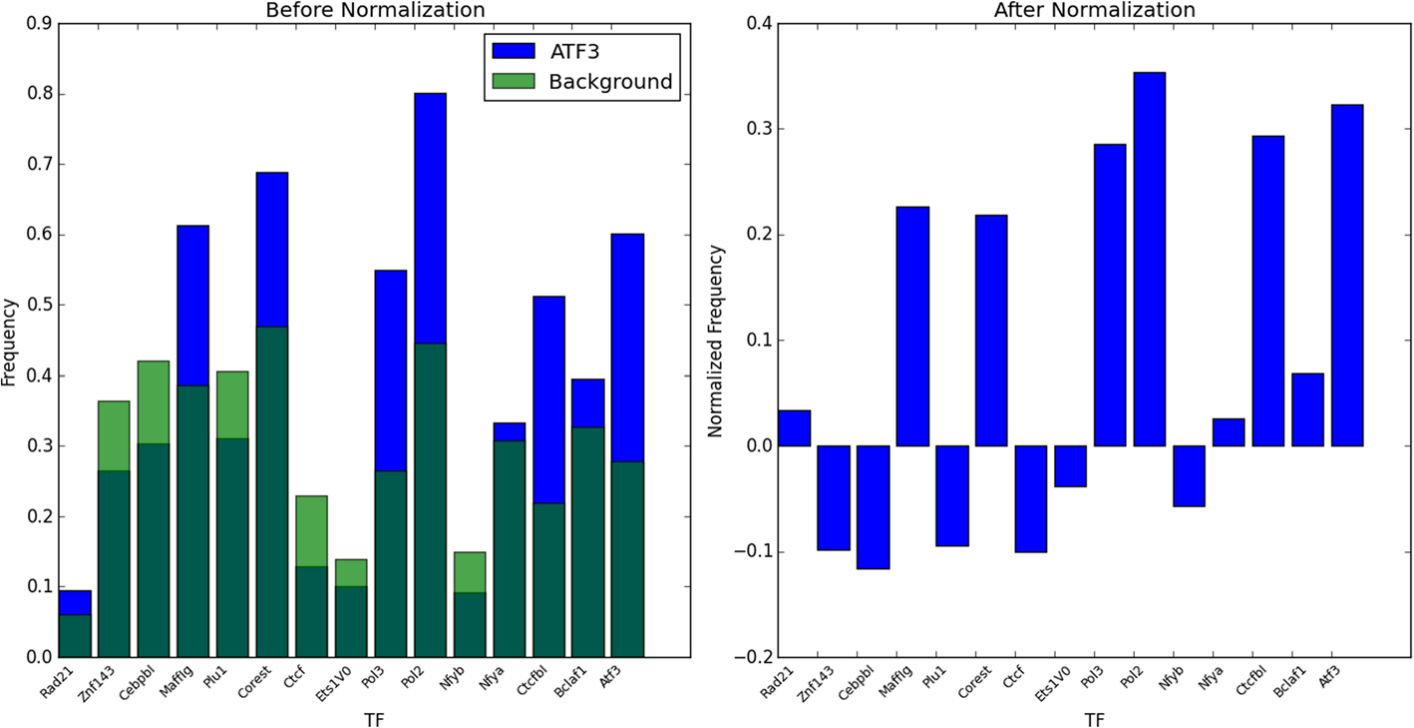
The first fifteen elements in the co-occurrence frequency vector for TF ATF3, shown as a bar graph, before and after normalization. Note how the pre-normalization frequencies of NFYA, ZNF143, and PLU1, which appear significant pre-normalization, are reduced post-normalization

Subtracting **f**_**all**_ from **f**_**t**_ ensured that each element in **f** ^′^_*t*_ represented only the magnitude of the TF-TF interactions, and not the background prevalence of that TF. Using subtraction for normalization penalizes the co-occurrence frequencies evenly; if the subtraction was substituted with division, high-frequency TFs such as CTCF would be overpenalized, while the co-occurrence of low-frequency TFs would be exaggerated. It also allows for negative frequencies, a factor which is utilized in the TFBS filtering described later. See Fig. 3 for an example of co-occurrence frequencies before and after normalization.

The adjacency matrix **A** is constructed by concatenating the normalized co-occurrence frequency vectors such that

$$\mathbf{A} = \left(\begin{array}{lll} {\mathbf{f}^\prime}_{T_{1} 1} & \cdots & {\mathbf{f}^\prime}_{T_{n} 1} \\ \vdots & \ddots & \vdots \\ {\mathbf{f}^\prime}_{T_{1} n} & \cdots & {\mathbf{f}^\prime}_{T_{n} n} \\ \end{array}\right) $$

and is used to create an undirected graph where normalized co-occurrence frequencies weight edges and TFs are nodes. For the ChIP-seq dataset, the graph contained 111 nodes with 6216 edges. For the ENCODE-motif dataset, the graph contained 585 nodes with 171,405 edges. For the ChIP-seq dataset, construction of the adjacency matrix required 3 min, 52 s on a Core i5-6300U CPU, using less than 5 GB of memory. For the ENCODE-motif dataset, construction of the adjacency matrix required 23 min, 44 s when using four cores in parallel and required less than 14 GB of memory.

### Comparing edge weight and motif similarity

An advantage of our method is its ability to detect interactions between TFs which are not based on binding motif similarity. That is, if a certain TF binds the genome combinatorially with other TFs at multiple sequences, a PWM matrix-based clustering method would fail to identify its interactions because of the TF’s weak association with a any particular sequence. Our proximity-based method, however, compares genomic positions rather than PWM matrices, and would therefore be able to detect such interactions.

To demonstrate that our method is capable of capturing TF interaction information beyond that represented by motif similarity, we compared the co-occurrence values derived by our method with the PWM similarities provided by the ENCODE-motif dataset. For each pair of TFs, we found the PWM similarity score within the ENCODE-motif dataset, averaging similarity scores whenever a given TF had multiple PWMs. We set up a simple linear regression, examining the extent to which these TF-TF PWM similarity scores predicted our method’s co-occurrence edge weights. We expected a low *R* ^2^, signifying that motif similarity explained only part of the TF-TF interaction information captured by our method. The results of this analysis are presented in the section “Motif co-occurrence provides more information than similarity alone” in the Results.

### Edge filtering using the FPR

Before the graph was clustered, its edges were filtered to remove edges with statistically insignificant weights. While the normalization procedure outlined above did involve subtracting a population mean from a sample mean, this sample was inherently non-random, as the binding sites associated with a TF are non-random. Parametric methods, then, could not be used to determine an ideal cutoff below which edges can be considered insignificant. Instead, an empirical, permuation-based method targeting a user-selected false positive rate (FPR) was employed. In this context, the FPR is the ratio of false positives, or insignificant edges wrongly considered significant (Type I errors), to the total number of truly insignificant edges [22].

In order to determine the edge weight cutoff for a given FPR, the adjacency matrix construction procedure was followed, but the co-occurrence matrices were replaced with dummy matrices. Each row of the dummy matrices was randomly generated, with its sparsity matching that of its overall genomic background frequency (its entry in **f**_**all**_). This created a situation where the overall prevalence of TFs was preserved, but their order throughout the genome, and therefore their proximity to other TFs, was randomly shuffled. All edge weights produced in these circumstances were therefore the result of random fluctuations rather than any real TF-TF associations. For both the ChIP-seq and ENCODE-motif datasets, this procedure was repeated 25 times, generating 308,025 and 8,555,625 dummy edge weights, respectively.

An edge weight threshold was then selected; any edge derived from the dummy matrices with weight greater than this threshold was then a false positive, and any with weight less was a true negative. Thresholds were selected to reach various FPR values, namely 0.01, 0.001, and 0.0001, and these thresholds were used to filter the graph, with any edges with weight lower than the the threshold removed. An FPR of 0.1 was also used for comparison purposes, to create a baseline graph with many false positive edges against which the three filtered graphs could be compared. This allowed us to assess whether filtering edges using the FPR degraded clustering performance. Using these FPRs, four new filtered graphs were therefore created for each dataset, which were subsequently clustered. See Table 1 and the “Results” section for a comparison of clustering at different FPR thresholds.

**Table 1:** Clustered graph metrics

	ChIP-seq				ENCODE-motif			
FPR	0.1	0.01	0.001	0.0001	0.1	0.01	0.001	0.0001
Nodes	111				585			
Edges Filtered	3032	3429	3973	4349	132141	146638	155684	160755
Pct. Edges Filtered	48.8	55.2	63.9	69.6	77.1	85.6	90.8	93.8
Clusters	10	11	10	12	51	52	54	51
Med. Nodes per Cluster	6	6	4.5	6	5	5	5	4
Max. Nodes per Cluster	36	25	37	25	135	143	129	139
Unclustered Pct.	18.0	13.5	13.5	15.3	0	0.342	1.19	3.42

### Comparison to protein-protein interaction data

To show that the TF-TF interactions found by our method are valid, we compared our TF-TF interaction data to the STRING protein-protein interaction database [23]. We first matched our TFs with entries in the STRING database, excluding data for any TF which could not be found in STRING. For the ChIP-seq dataset, 4 of 111 TFs (3.6%) could not be matched; for the ENCODE-motif dataset, 45 of 585 TFs (7.7%) could not be matched. A STRING adjacency matrix was then constructed with the same structure as the TF adjacency matrix. Each element *i*,*j* in the STRING adjacency matrix represented whether or not (1 or 0) an interaction between TF *i* and TF *j* was found in the STRING database. The STRING adjacency matrix was then compared with the filtered adjacency matrices produced by our method; a true positive was counted if the two corresponding entries in each matrix were both nonzero. True positives, false positives, true negatives, and false negatives were counted and used to calculate the precision, recall, and F-score of our predicted interactions when compared to the STRING database. For a more in-depth discussion of these metrics, see the section “Filtering of putative TFBSs”. We expected that our predicted interactions would correspond to some extent with the STRING database. However, because our data is TF-specific and derived from TF proximity in reference to the genome rather than the pathways, ontology, and experimental data that underlie STRING interactions, we expected a large number of novel and differing predictions as well.

STRING further splits its interaction scores into seven evidence categories: Co-expression, Experiments, Database, Text-Mining, Neighborhood, Fusion, and Co-occurrence. Given these diverse data sources, we also explored if the interactions detected by our method were significantly more enriched in one of the categories when compared to the others. The Co-expression and Experimental categories are the most relevant to our analysis. The Co-expression score describes protein interactions in terms of consistent appearance in expression studies, as would be expected of interacting TFs, and the Experiments category describes interactions that have been confirmed in a lab rather than predicted or inferred. The Neighborhood, Fusion, and Co-occurrence evidence channels are least relevant, as they are designed for use in bacteria and archaea protein-protein interaction analysis [23]. Therefore, significant enrichment of our method’s STRING matches in the Co-expression and Experimental categories would provide support to our predictions.

### MCL clustering

We chose the Markov Clustering Algorithm (MCL), a graph paritioning algorithm, to cluster the filtered networks. Traditionally, hierarchical clustering, rather than graph partitioning, has been used for similar tasks, but we believe it bears significant downsides as opposed to a true graph partitioning algorithm such as MCL [24]. First, while hierarchical clustering’s tree output provides an intuitive representation of some inter-cluster relationships, “how far up” in the tree to call clusters distinct is not clear. Additionally, hierarchical clustering does not allow nodes to belong to more than one group without dramatically increasing the size of the group. Graph partitioning algorithms simply cluster nodes while preserving the structure of the graph, allowing for more relationships between nodes and clusters and better exploration. As a result, we chose a partitioning algorithm over a hierarchical clustering algorithm.

In a review by Brohee and van Helden, the MCL algorithm was shown to be better suited to clustering protein-protein interactions than three other graph partitioning algorithms, and was therefore chosen for this similar task [25]. We used the MCL algorithm as part of the ClusterMaker suite within graph visualization software Cytoscape for our analysis [26, 27]. The MCL algorithm attempts to partition graphs into clusters by simulating random walks among nodes, where the likelihood of following a given path is based on edge weight. The algorithm then trims paths with the lowest traversal likelihood and repeats the process. For a full discussion of the algorithm, we refer the reader to Van Dongen’s original publication [28].

MCL depends on three parameters, a granularity parameter, pruning threshold, and an iteration limit; the algorithm’s performance is relatively insensitive to all three. We adjusted only the first, choosing it empirically based on number of clusters produced. Regardless of the dataset or filtering level, the best performing granularity parameter was simple to acquire and always fell between 2 and 5.

### Filtering of putative TFBSs

To demonstrate how the graph structure could be used to improve the accuracy of TFBS searching, we performed filtering of putative, motif-based TFBSs for the transcription factor ATF3 (the *target* TF). Here, our method was based on the assumption that a putative TFBS is more likely to be a true positive if it is found near its co-occurring counterparts. We first generated the graph from the ENCODE-motif dataset, but left it unfiltered and did not remove negative edges. Negative edges were helpful in this situation, as the more negative the edge was, the *less* likely the TF was to be found with the target.

The ChIP-seq dataset was not used for filtering, as its data was the “ground truth.” While the motif PWMs in the ENCODE-motif dataset are derived from ChIP-seq data, the individual putative TFBSs within the ENCODE-motif dataset are found by scanning the PWMs across the genome and checking for matches. As a result, the ENCODE-motif TFBSs are putative, and contain a large number of false positives. In the method below, no information from the ChIP-seq dataset is used to filter the ENCODE-motif dataset, and therefore, the ChIP-seq data provides a ground truth with which to compare our filtering results.

TFBS searching using motifs can be seen as an information-retrieval problem. Information retrieval attempts to maximize the number of relevant “documents” in a pool of retrieved documents [29]. In this case, retrieved documents were the putative TFBSs, and relevant documents were putative TFBSs which matched actual (from ChIP-seq) TFBSs. The performance of information-retrieval systems is often evaluated in terms of recall, precision, and the *F*-score.

Recall, or sensitivity, is the fraction of relevant documents that are successfully retrieved - the fraction of actual ChIP-seq TFBSs marked by putative motif TFBSs. To determine the recall of the putative TFBSs, a 1000 base-pair window was created around each actual (ChIP-seq determined) ATF3 binding site. The number of actual TFBSs with putative (motif) TFBSs within their surrounding window were considered true positives; this sum was divided by the total number of actual TFBSs to produce the recall.

Precision, also known as positive predictive value, is the fraction of retrieved documents that are relevant - the fraction of putative TFBSs that correspond to true ChIP-seq TFBSs. To determine the precision of the putative ATF3 TFBSs, the putative TFBSs were first merged, such that any overlapping putative TFBSs were condensed into one larger TFBS. Then the previous procedure was repeated. In this case, however, the 1000 base-pair windows were placed around the putative TFBSs and the divisor was the total number of putative ATF3 TFBSs.

The *F*-score, the harmonic mean of precision and recall, was also calculated as an overall measure of TFBS searching performance.

To maximize precision with a minimal reduction in recall, false putative TFBSs needed to be filtered out without removing those truly corresponding to ChIP-seq TFBSs. To accomplish this, a “sum-score” was assigned to each putative ATF3 binding site. A 1000 base-pair window was created around each site, and all neighboring TFs within this window were recorded. The score, then, was the sum of all edges from ATF3 to its neighbors within the window. If ATF3 was not often found with a neighbor at a given TFBS, the score would be decreased due to a negative edge, and the inverse also held. Thus, if a window contained many highly co-associated TFs, the score was maximized. A threshold was chosen, and all putative TFBSs with scores less than this weight were eliminated. Precision, recall, and the *F*-score were calculated on the filtered set. To produce a precision recall curve (a close relative of the binary classification reciever operating curve, see [30]) the threshold was adjusted from its minimum (such that no putative TFBSs were removed) to its maximum (such that all TFBSs were removed), and the precision, recall, and *F*-score were recorded at each point.

We compared the precision-recall curve and maximum *F*-score from our sum-score with those of three alternate methods. The first removed the same number of TFBSs as the sum-score threshold, but did so randomly, testing if any increase in accuracy was due simply to reduction in the number of TFBSs returned rather than any association between TFs. The second was a score computed simply as the *number* of neighboring TFBSs in each window; it tested if any increase in accuracy was due to the raw number of neighboring TFs (indicating a possibly highly-active regulatory region). Finally, we calculated a modified sum score, where each window’s score was normalized by the number of TFs within it; this tested whether co-association alone could out-perform the combination of number of neighbors and co-association which the unmodified sum-score embodied.

## Results

### A low FPR yields discrete TF clusters

For both datasets, each FPR level produced a clustered graph, each of which is summarized in Table 1. For each graph, the first cluster was always significantly larger than the others; this “omnibus” cluster was undesirable as it prevented its constituents from joining other, more interpretable clusters. On the other hand, a low median nodes per cluster indicated that possible interactions were being missed. There were also some nodes not assigned to any cluster in each graph, though it was not clear if these nodes were unclustered because they truly did not belong to any clusters or because too many of thier edges were removed as part of the filtering process. Thus, the best performing graph for each dataset balanced a low FPR, relatively low unclustered percentage, intermediate median nodes per cluster, lower maximum nodes per cluster, and a higher number of clusters.

For the ChIP-seq dataset, FPR 0.01 offered this best balance, while for the ENCODE-motif dataset, FPR 0.001 was the best clustered graph. For both datasets, the median nodes per cluster was manageable, with most nodes congregating in small, interpretable clusters rather than large ones. Between the two datasets, the ratio of clusters to nodes and max nodes per cluster to nodes were similar, but upon visual inspection, the ENCODE-motif dataset appears to perform better, with more clusters outside of the large “omnibus” cluster. This is most likely due to the fact that the ENCODE-motif dataset has more nodes to cluster and therefore more clusters to produce. While the images of the entire graph are too large to include in this manuscript with sufficient detail, see the Additional files 1 and 2 section for Cytoscape graph files of the both the ChIP-seq and ENCODE-motif datasets.

When comparing to the high false-positive (FPR=0.1) graphs, we see that good clustering performance was still achieved at low FPRs. We saw that both the ChIP-seq and ENCODE-motif datasets performed equally to the baseline high-FPR (0.1) in terms of clusters, median nodes per cluster, and maximum nodes per cluster, but differed in terms of unclustered percentage. For the ENCODE-motif dataset, we observed the intuitive increase in unclustered nodes as the FPR, and therefore the number of edges filtered, increased. As FPR increased and more edges were cut, more nodes would become disconnected and therefore unclustered.

The ChIP-seq dataset, however, showed the opposite trend, with the high-FPR (less edges filtered) dataset having more unclustered nodes. This is due to the low percentage of edges filtered at this FPR. The 0.1 FPR ChIP-seq graph filters only 48.8% of the edges, while the ENCODE-motif graph still filters 77.1%. We observed that the MCL algorithm failed to adequately cluster the data when there were too many edges included, leaving larger “omnibus” clusters and more unclustered nodes. The FPR of 0.1 for the ChIP-seq dataset, then, failed to trim enough edges, causing an increase in the number of unclustered nodes.

In this way, FPR acts as a tuning parameter. Increasing it reduces noise at the cost of disconnecting nodes and increasing unclustered nodes. Decreasing it increases noise while allowing more nodes to be clustered, up to the point that too few edges are filtered and MCL fails to adequately cluster the nodes.

### TF clusters agree with known TF-TF interactions

Many of the ChIP-seq and ENCODE-motif datasets’ clusters embodied known TF-TF interactions, lending credence to our method’s accuracy. The ChIP-seq FPR 0.001 graph includes the experimentally known SM3A-CTCF, JUN-FOS, TAL1-EGR1, JUN-NFY, STAT1-GATA1, and ELK1-STAT2 interactions, among others [31–36].

Many clusters from the ENCODE-motif dataset group the different motifs from the same family, such as the DMRT family in Fig. 6. This is expected, as motif PWMs within the same family would be expected to be highly similar. Other clusters, however, include both intra- and extra-familial interactions, and these contain the known CREB-ATF, BACH-NFE2, NFIL3-HLF, NR2F-HNF (see Fig. 6), and YY-SRF interactions, among others [37–41].

When compared to the STRING protein-protein interaction database, the ChIP-seq dataset has a recall of 0.4342, a precision of 0.3736, and an F-Score of 0.4016, with the FPR=0.01 graph performing best. The ENCODE-motif dataset has a recall of 0.2051, a precision of 0.2282, and an F-Score of 0.2161, with the FPR=0.01 graph again performing best. Because our method finds TF-TF interactions based on genomic colocation and is entirely focused on transcription factors, while STRING is focused on all protein-protein interactions and derives its interactions from very diverse data sources, it is expected that our method would produce many novel predictions when compared to STRING. Even so, 37% (over 4000) and 22% (over 75,000) of the TF-TF interactions predicted by our method were also contained within the STRING database for the ChIP-seq and ENCODE-motif datasets, respectively, and our precision and recall values correspond to those of several other *in silico* protein-protein interaction prediction methods [11, 42–47]

**Fig. 4:**
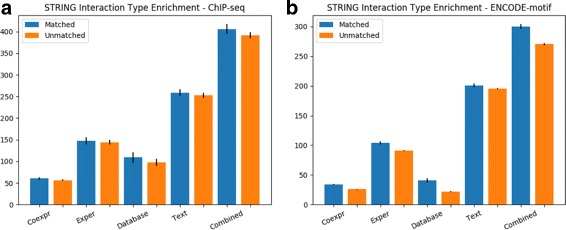
A comparison of STRING evidence category enrichments between STRING interactions which matched our predicted TF-TF interactions and those that did not, for (**a**) the ChIP-seq dataset, and (**b**) the ENCODE-motif dataset

For the ChIP-seq and ENCODE-motif datasets, we found that our method identified TF-TF interactions which were significantly (*p*<0.05 and *p*<0.001, respectively) more enriched in the Co-expression evidence category when compared to STRING interactions which were not predicted by our method. This indicates that our method preferentially identifies interactions containing TFs that are consistently present in the same cell at the same time, as would be expected of interacting TFs. The ENCODE-motif dataset is also significantly enriched in the Experimental, Database, and Text-mining categories (*p*<0.001 for each). The Experiment and Database enrichment is especially important, as it provides evidence that our method preferentially captures interactions which have been experimentally derived. Figure 4 compares the evidence category enrichments for out method’s TF-TF interactions.

**Fig. 5:**
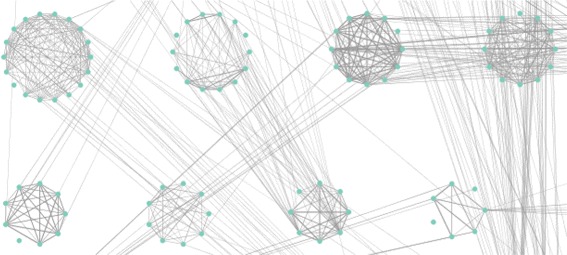
A zoomed-out portion of the ENCODE-motif clusters, with some inter-cluster edges shown, demonstrating how entire clusters can be highly connected to some clusters but not others and raising the possibility of cluster-cluster interactions

The presence of many experimentally validated TF-TF interactions among the clusters, a degree of correspondence with previous protein-protein interaction data similar to other *in silico* methods, and enrichments in experimentally-derived interaction evidence categories leads us to conclude that our method provides a cheap, high-throughput window into identifying TF-TF interactions on a putative basis. Also, unlike experimental assays which are blind to the larger framework the complexes they detect may participate in, our method preserves inter-cluster edges, leaving cluster-cluster interactions (see Fig. 5) or single-TF many-cluster interactions free to be explored. Additionally, while the clusters assigned by our method are putative, we believe the accuracy, cheapness, and speed of our method allows it to be used as a springboard by which to direct future research, allowing experimental investigators to start with potential TF-TF interactions instead of “from scratch.”

**Fig. 6:**
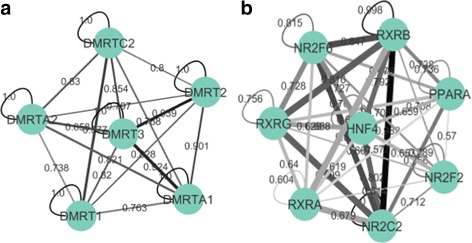
Two clusters from the ENCODE-motif dataset, with edge thickness representing the co-occurrence frequency edge weight generated by our method, and edge color (light gray to dark black) representing PWM similarity (used in similarity-based methods). In (**a**), all of the TFs are from the same family (DMRT), and therefore have high levels of motif similarity (all the edges are dark). Our method is able to group them together because they co-occur often. In (**b**), the grouping has both intra- and extra-familial connections, with some TFs having dissimilar (light gray) PWMs. Many of these interactions could not be picked up a motif similarity-based method

### Motif co-occurrence provides more information than similarity alone

A potential advantage of our method is its ability to detect TF interactions outside the realm of motif PWM similarity. The regression outlined in “Comparing edge weight and motif similarity” showed *R* ^2^ to be 0.262, indicating that motif similarity accounted for only 26.2% of the variance in normalized edge weight. This implies that motif similarity does not automatically equal motif co-occurrence, especially when co-occurrence is normalized against total background occurrence as it has been in our method.

Practically, this can mean the difference between spotting a TF-TF interaction and missing one. In Fig. 6, our method grouped the TFs together regardless of the fact that their PWM similarities (signified by edge darkness) are largely inconsistent, with some interactions within the cluster having highly similar motifs and others weak motif similarity. A similarity-based method would fail to group the experimentally validated HNF-NR2F2 interaction found in this cluster due their PWM dissimilarity (lighter gray edge in Fig. 6), but our method was able capture the interaction because they co-occur often (thicker edge in Fig. 6).

### Filtering of putative TFBSs significantly improves accuracy

Without filtering, the recall of ATF3’s putative TFBSs was 0.277, while the precision was only 0.0053, giving an *F*-score of 0.0104. Our method achieved a maximum *F*-score of 0.0725, an increase of nearly seven times the unfiltered *F*-score, and increased precision by a factor 12.6 to 0.0667. At the same time, recall at the maximum F-score only decreased by a factor of 3.4 to 0.0795. Additionally, if the recall is held at the original, unfiltered level of 0.277, the normalized sum-score doubles the unfiltered precision, at 0.0104. It should be noted that this was achieved in a completely unsupervised manner, with ground truth experimental ChIP-seq data used only to determine after-the-fact accuracy.

**Fig. 7:**
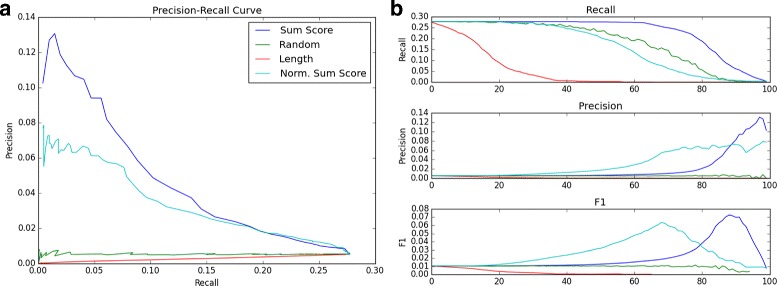
**a** The precision-recall curve for TFBS filtering of ATF3. A more gradual decrease in precision with the increase in recall signifies better performance. “Length” indicates the score based on the number of neighboring TFBSs within each window. **b** The recall, precision, and *F*-score plotted as the cutoff (number of filtered TFBSs) increases

Several interesting observations were taken from Fig. 7. We found that the non-normalized sum score performed the best compared to the other scores evaluated, achieving the slowest drop in recall, the greatest increase in precision, and the best overall precision-recall curve. Both the non-normalized and normalized sum-scores performed much better than the random-removal null metric, indicating that the motif co-occurrence used to create our score truly captures information that allows it separate true putative TFBSs from false ones.

Additionally, the number of TFs in each TFBS window performed significantly *worse* than both the random removal and sum-score, with no increase in precision and a faster decrease in recall. Upon further investigation, we found that number of neighboring TFBSs was actually strongly *negatively* correlated with the sum-score (*R*=−0.81). We flipped the thresholding to account for this, such that the cutoffs went from high number of neighbors to low (reflected in the corresponding curve in Fig. 7), but the performance was still worse than random. This meant that “quality” of neighboring TFs was more important than “quantitity” when filtering; as the number of neighboring TFs increased, more erroneous TFs with negative edge weights crept in, decreasing the score.

At the same time, however, the non-normalized sum-score performed marginally better than the normalized sum-score, meaning that removing the effect of the number of neighboring TFs in each window altogether was detrimental rather than helpful. We believe this is due to a “boosting” effect which the non-normalized sum-score allows. In a situation where a putative TFBS not only has frequently co-occurring neighbors but the added benefit of *many* of them, the non-normalized score takes this into account while the normalized cannot, giving the non-normalized score a slight performance advantage.

While the normalized sum-score performed slightly worse in terms of raw F-score, it cannot be discounted, as the normalized score achieved only slightly lower metrics while maintaining a lower cutoff value. This meant that the normalized score left more TFBSs in the filtered set, which would be ideal if further processing on the filtered set was desired.

From the above results, we can conclude that on a proof of concept basis, our unsupervised co-occurrence based method can significantly increase the accuracy of motif searching, capturing information beyond that given by density of TFBSs or motif similarity (see previous section). Moreover, this filtering method requires no supervised training with experimental data. The success of this co-occurrence method filtering further lends credence to the clustering results described above; if co-occurrence captures relationships between TFs to the extent that it can veritably improve TFBS searching, the clusters based on those same co-occurrences are likely to incorporate true relationships.

## Discussion

The TF regulatory framework is essential to cellular regulation and human health but is difficult to understand, with interactions between TFs and the genome as well as between TFs themselves. While established methods exist to locate where TFs bind the genome, the clustering and interaction of TFs themselves is largely unexplored. Many clustering methods use motif PWM similarity, which leaves out interactions not based on sequence affinity, and methods which use proximity data from ChIP-seq or motif analysis have not been applied to true TF clustering. In this study, we presented a proximity-based graph clustering solution for the identification of TF clusters.

We used TF co-occurrence data from two TFBS datasets to develop graphs, which were then filtered using an empirical FPR thresholding method. MCL was applied to the filtered graphs, producing many distinct, manageable clusters, with the motif-based dataset performing best. Good clustering performance was achieved even at very low FPRs where as much as 94% of graph edges were filtered out. FPR represented a trade-off parameter; a decreased FPR would decrease the maximum nodes per cluster and increased the total number of clusters, but this increased the number of orphaned, unclustered TFs.

Furthermore, we believe that our results provide evidence that clusters produced by our method are accurate and correspond to biological realities. First, many experimentally known TF-TF interactions were identified within our graphs’ clusters, and our graphs correspond well to the protein-protein interactions found in the STRING database, indicating on an empirical basis that our high-throughput, one-pass method is capable of accurate cluster assignment. Our use of the MCL algorithm leaves inter-cluster edges intact, which more closely mirrors the biological reality of a highly-connected, complex regulatory system and allows for further exploration of TF-TF and cluster-cluster interactions.

Second, the information contained within the graph structure was able to significantly improve the accuracy of motif-based TFBS searching for the TF ATF3 when compared to ground truth ChIP-seq data, which indicates that the co-occurrence of TFs with one another has direct biological relevance. Our filtering was based on the simple assumption that if a given TF tends to co-occur with a set of other TFs, a putative binding site without the frequently co-occurring TFs is likely to be a false positive. The unfiltered, unclustered graph was used to generate scores for each putative TFBS, and TFBSs with scores below a certain threshold were filtered out. The precision-recall curves for the graph-based scores significantly outperformed those based on number (rather than identity) of co-occurring TFs as well as a random control. The TF-TF relationships identified by our method must therefore have a biological basis, as they bring the accuracy of motif-based TFBS searching closer to that of the ChIP-seq biological reality.

**Fig. 8:**
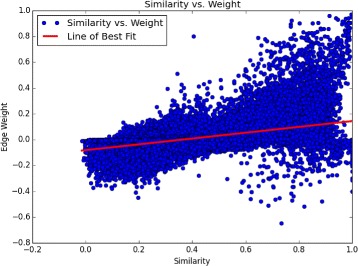
Motif similarity poorly predicts the edge weight used in our analysis, indicating that our method can capture interactions that extend beyond motif similarity

Finally, by regressing motif PWM similarity with the edges of our graph, we demonstrated that our method captures information beyond PWM similarity in the ENCODE-motif dataset. PWM similarity only accounted for 25% of the variance within our TF-TF associations, allowing for TF-TF interactions to be found where they otherwise would be missed (Fig. 8).

A primary limitation of our method is that while it identifies many TF interactions in one pass, including many which have been already experimentally confirmed, it does so on a putative basis, and it does not offer a quantitative level of likelihood for the clusters it produces. Our method, however, is not meant to replace experimental investigation or confirmation of TF clusters. Instead, it is meant as a tool to quickly and easily explore TF-TF interactions on a putative basis. Additionally, when used with ChIP-seq datasets taken from different cell types or under different cellular conditions, it allows for direct visualization of how TF clusters, and therefore the regulatory environment, change from cell to cell and condition to condition.

## Conclusions

Transcription factors form a complex regulatory network which is crucial to human health but is difficult to understand. Methods such as ChIP-seq and motif searching can identify where a TF is likely to bind, but TFs also interact among themselves in ways that are largely unexplored. To address this, we used TF co-occurrence data to develop filtered, normalized graphs, with edges representing the degree of association between TFs. We clustered these graphs using the MCL algorithm, which produced many distinct clusters with known TF-TF interactions while preserving inter-cluster edges. We also demonstrated that our proximity-based co-occurrence method captures information, and therefore TF interactions, that PWM similarity methods cannot. The biological correspondence of our graph output was also demonstrated when we compared it to protein-protein interactions in the STRING database, as well as when we used the graph structure to filter putative TFBSs for ATF3, significantly reducing the number of false positives and increasing accuracy. Going forward, this method can be applied to examine how TF clusters change between cellular conditions and cell types, opening doors into the “second dimension” of TF regulatory dynamics.

## Additional files

Additional file 1 **chip_graphs.cys.** Cytoscape file of the ChIP-seq dataset graph and its clustered graphs as described in the Results section. (CYS 318 kb)

Additional file 2 **motif_graphs.cys.** Cytoscape file of the ENCODE-motif dataset graph and its clustered graphs as described in the Results section. (CYS 9216 kb)
